# Surgical Outcomes Following First Metatarsophalangeal Joint Replacement: A Cohort Study

**DOI:** 10.3390/jcm15166170

**Published:** 2026-08-09

**Authors:** Hamza Idrees, Liviu-Coriolan Misca, Rehan Gul

**Affiliations:** 1 The Queen Elizabeth Hospital Kings Lynn NHS Foundation Trust, Gayton Rd, King’s Lynn PE30 4ET, UK; 2 Barts and the London School of Medicine and Dentistry, Garrod Building, Turner St, London E1 2AD, UK; 3 Department Orthopaedics and Traumatology, Cork University Hospital, T12 DC4A Cork, Ireland; rehangul@hotmail.com; 4 Orthopaedics Department, University of Medicine and Pharmacy Victor Babes, Timisoara, 300041 Timisoara, Romania; 5 Department Orthopaedics and Traumatology, University College Cork, T12 K8AF Cork, Ireland

**Keywords:** hallux rigidus, metatarsophalangeal joint, arthroplasty, hemiarthroplasty, joint replacement, AOFAS, functional outcomes

## Abstract

**Background/Objectives**: First metatarsophalangeal (MTP) joint arthritis is a debilitating condition often treated with arthrodesis or joint replacement. However, the effectiveness of MTP joint replacement has not been thoroughly researched. This study aimed to evaluate functional outcomes and complication rates following first MTP joint replacement over a maximum seven-year follow-up period. **Methods**: A retrospective cohort study of 60 patients (68 toes) who underwent MTP joint replacement was performed. Data were extracted from medical records and anonymised prior to analysis. The primary outcomes measured included functional improvement and pain relief (AOFAS). **Results**: Findings indicate significant improvement in mean active and passive range of motion (ROM), with a mean active ROM of 35 degrees and a mean passive ROM of 45 degrees from a pre-op ROM of 0–10 degrees. The overall complication rate was 20.58%, with 10 cases of persistent stiffness but no pain and four cases of continued post-operative pain that necessitated MTP arthrodesis. Patients reported a mean post-operative AOFAS Hallux MTP-IP score of 88, improving from a pre-op score of 35. **Conclusions**: Results suggest that first MTP joint replacement is associated with significant pain relief and functional improvement with acceptable complication rates over the study period.

## 1. Introduction

Hallux rigidus (HR) is a common condition characterised by degenerative arthritis affecting the joint at the base of the first MTP joint. It typically affects 2.5% of individuals aged 50 and older and is the most prevalent type of foot joint arthritis. It can occur bilaterally in patients with a family history of HR but can also occur unilaterally. HR is characterised by pain and a reduced range of motion. The aetiology of hallux rigidus is both multifactorial and not fully understood. Possible reasons include recurrent microtrauma, elongated or elevated first metatarsal (metatarsus primus elevates), hyperpronation of the foot, and a flat or chevron-shaped metatarsal head. Aberrant joint loading and systemic inflammatory diseases like rheumatoid arthritis or gout may also play a part. Genetic predisposition is also acknowledged, as bilateral disease is more prevalent in patients with a positive family history, whereas unilateral disease is more often associated with prior trauma to the first MTP joint. The disease has a gradual progression from mild dorsal osteophyte formation, through severe destruction of the joint, which leads to extensive articular cartilage loss, subchondral sclerosis and global joint stiffness. Classification instruments, in particular the Coughlin and Shurnas grading scale, are used to determine different levels of disease severity ranging from grade 0 (normal joint) to grade 4 (severe degenerative change in the joint with significant loss of motion and lasting pain), and these classifications are commonly used to guide conservative and surgical choices.

Treatment involves non-surgical interventions, such as adjusting shoes and using orthotics to restrict MTP movement. Conservative measurements also include activity modification and weight reduction in appropriate patients. The use of rigid-soled or rocker-bottom footwear and carbon fibre Morton’s extension insoles has also aided in pain relief. Non-steroidal anti-inflammatory drugs (NSAIDs) and intra-articular corticosteroid or hyaluronic acid injections are also effective interventions in early disease and aim to reduce inflammation and painful dorsiflexion at the first MTP joint, in hopes of delaying surgical intervention. However, in patients with advanced disease, conservative management often proves insufficient, with persistent pain and functional limitation prompting surgical referral. Surgical techniques for individuals with mild arthritis include removing excess osteophyte growth (cheilectomy), periarticular osteotomy, and interposition arthroplasty, which have shown favourable results for patients with grade 1 and 2 hallux rigidus. Arthrodesis of the first MTP joint has been the primary focus of operative management and is considered the gold-standard strategy for patients with advanced arthritis. However, MTP joint replacement is a great alternative to reduce pain and preserve the range of motion in patients suffering from foot joint arthritis. The growing interest in joint-preserving procedures has been driven by patient demand for retained mobility, particularly among younger and more active individuals who wish to continue participation in recreational and occupational activities which require dorsiflexion of the great toe. Arthrodesis, despite its effectiveness in alleviating pain, almost completely eliminates the motion of the first MTP joint. This can compromise activities of daily living such as running, squatting, climbing stairs, and wearing certain types of footwear. In light of this, hemiarthroplasty has emerged as an attractive alternative for selected patients, offering the dual benefits of pain reduction and joint mobility preservation. This study aims to evaluate the outcomes of first MTP joint hemiarthroplasty in a single-surgeon cohort of patients with hallux rigidus with a focus on pain management, range of motion, and functional outcomes [1,2,3,4,5].

## 2. Materials and Methods

This is a retrospective cohort study of patients who underwent first MTP joint hemiarthroplasty. Ethical approval was obtained from the Clinical Research Ethics Committee (CREC) prior to the commencement of data collection. All data were extracted from medical records and anonymised prior to analysis; no re-identification key was retained. All data analysis was conducted within the participating institutions, and only aggregated results were shared among collaborators. The study was conducted in accordance with the principles of the Declaration of Helsinki and adhered to local data protection legislation, including compliance with the General Data Protection Regulation (GDPR). Informed consent for the surgical procedure was obtained from all patients preoperatively, and patients were informed at the time of consent that anonymised clinical data might be used for audit, quality improvement, and research purposes. Given the retrospective and fully anonymised nature of the analysis, a waiver for additional research consent was granted by the ethics committee.

Inclusion criteria: Patients diagnosed with hallux rigidus between 2015 and 2020, with no deformity and continuous joint pain for at least six months despite conservative management. According to Coughlin’ classification, patients suffering from HR stage 2–4 underwent hemiarthroplasty of the first MTP joint with a cobalt-chrome articular implant.

Exclusion criteria: Patients diagnosed with HR with deformities, patients with HR stage 1, and patients with a minimum follow-up of less than one year. Additional exclusion criteria included active local or systemic infection and inflammatory arthropathies such as rheumatoid arthritis or psoriatic arthritis affecting the first MTP joint, as well as significant peripheral vascular disease preventing safe surgery, severe osteoporosis compromising implant fixation, and previous surgery on the ipsilateral first MTP joint.

The study included 60 patients (68 first MTP joints) who met the inclusion criteria. A single surgeon performed all surgeries.

All patients underwent surgery under general anaesthesia; the use of tourniquet created a bloodless field. Before sedation, prophylactic antibiotics were administered to each patient. The procedures involved a dorsal approach, and all patients were treated with The Hemi CAP system from Arthrosurface Inc. in Franklin, MA, USA. This system utilises a contoured articular prosthetic with cobalt-chrome and titanium components that securely fixate to the metatarsal head through a taper interlock mechanism. Additionally, patients underwent capsular and sesamoid release. The surgical technique was standardised throughout the study period to minimise operator variability. Following a longitudinal dorsal incision on the first MTP joint, careful dissection was performed to avoid damage to the dorsomedial cutaneous nerve. A capsulotomy was carried out and the joint was exposed. Dorsal osteophytes were excised using an oscillating saw, and a generous cheilectomy was performed to remove proliferative bone from both the metatarsal head and the proximal phalanx if indicated. The articular surface of the metatarsal head was next prepared in accordance with the manufacturer’s instructions, with a guide wire inserted perpendicular to the long axis of the metatarsal. Sequential reaming was performed to accommodate the Morse taper screw, into which the cobalt-chrome articular cap was later inserted. Correct implant positioning and alignment were confirmed by intraoperative fluoroscopy. Capsular and sesamoid release was performed to optimise postoperative range of motion, and the wound was closed in layers with absorbable sutures for deeper tissues and non-absorbable sutures for skin.

Post-operatively, patients were placed in a stiff-soled postoperative shoe and encouraged to mobilise with full weight-bearing as tolerated from day one. Active and passive range of motion exercises were commenced within 48 h of surgery to prevent stiffness and adhesion formation. Patients were referred for physiotherapy for structured rehabilitation. Patients were instructed to elevate the foot as often as possible in the first two weeks to reduce swelling and were prescribed simple analgesics with stronger opioids reserved for breakthrough pain. Sutures were removed during the two-week post-operative review. Post-operative follow-up included evaluation of pain scores and range of motion assessment based on clinical records. The lead surgeon conducted a range of motion assessment, and follow-up X-rays (lateral and AP) were taken to confirm the positioning of implant components. Patients were typically reviewed at six weeks, three months, six months, twelve months and annually thereafter. Clinical evaluation at each appointment included assessment of pain at rest and on activity, along with assessment of dorsiflexion and plantarflexion at the first MTP joint. Wound healing was also observed in the initial postoperative period, and footwear and gait were also reviewed. Radiographic assessment was done at each follow-up to identify any evidence of implant loosening, periprosthetic lucency, or progressive degenerative changes at adjacent joints.

The AOFAS Hallux MTP-IP was employed to evaluate the patient’s pre- and post-operation. Initially published in 1994 in Foot and Ankle International, the AOFAS Hallux Metatarsophalangeal–Interphalangeal Rating System is a standardised tool for evaluating the hallux and first metatarsal. This system combines subjective patient-reported pain with objective physician assessments of alignment [6]. It includes three main domains: pain (40 points), function (45 points) and alignment (15 points), for a total score of 100 points at maximum, which represents a pain-free, fully functional and well-aligned joint. Although the AOFAS scoring system has been subject to criticism for its limited psychometric validation, it remains one of the most widely used systems for measuring outcomes in foot and ankle surgery and serves as a useful basis for comparison across published studies [7,8,9,10].

Statistical analyses were performed using IBM SPSS Statistics (Version 3.5 V29.0, IBM Corp, Armonk, NY, USA). Normality of continuous variables was assessed using the Shapiro–Wilk Test. Comparisons between preoperative and postoperative AOFAS Hallux MTP-IP scores and ROM were performed using independent Student’s *t*-tests. Comparison amongst patients with different ages of hallux rigidus (Coughlin grades 2–4) was performed using one-way analysis of variance (ANOVA). All statistical tests were two-sided, and a *p*-value < 0.05 was considered statistically significant.

## 3. Results

This study encompassed 60 patients (68 toes) who underwent arthroplasty of the first MTP joint due to hallux rigidus. The patient cohort consisted of 30 females and 30 males, with an average age of 49.1 years at the time of surgery. The majority of patients presented with hallux rigidus between stages 2 and 4. This is consistent with the demographic typically considered for joint-preserving procedures, where preserving mobility is prioritised over the more predictable but motion-eliminating results of arthrodesis. Based on clinical records reviewed, the mean follow-up period was 3.5 years with a maximum of 7 years, during which the primary surgical outcomes assessed included range of motion and pain relief. There was statistically significant improvement in postoperative clinical outcomes. Mean active ROM improved to 35°, while mean passive ROM improved to 45°, compared with the preoperative 0–10° (*p* < 0.05 for all comparisons). Patients also demonstrated a statistically significant improvement in AOFAS Hallux MTP-IP score from 35 preoperatively to 88 postoperatively (two-sided *p* < 0.05) (Table 1).

The even distribution of male and female patients within the cohort enabled a balanced assessment of outcomes across both sexes. The subgroup analysis did not reveal any statistically significant differences in postoperative AOFAS Hallux MTP-IP scores or range of motion (two-sided *p* < 0.05) between both genders.

AOFAS Hallux MPT-IP scores range from 0 to 100, with 100 indicating a healthy joint. It can evaluate the first metatarsal, MTP joint, and associated phalanges, making it useful for conditions like hallux valgus, MTP arthrosis, and various toe deformities and injuries.

This system was used to assess the patients, revealing a notable improvement in mean AOFAS score from 35 preoperatively to 88 postoperatively (two-sided *p* < 0.05), indicating favourable outcomes. Improvements were observed across all three AOFAS domains, with the most apparent changes seen in the pain and function subscales, reflecting subjective as well as objective outcomes. Patients experienced relief from pre-operative pain, with most only feeling occasional mild discomfort in prolonged or strenuous activity at final follow-up. Improvement in functionality resulted in improved ease in walking, climbing stairs, and a return to physical activities such as cycling and golf within three to six months post-surgery. Moreover, 14.70% (10 toes) of patients experienced stiffness post-op but reported no pain, while only 5.88% (4 toes) required arthrodesis post-arthroplasty due to persistent pain, resulting in a 94.12% survival rate for implants in patients who underwent their first MTP joint replacement (Table 2).

Notably, patients could resume wearing wide-toe box shoes within 24 h and driving within three weeks post-surgery. Patients’ average return to work occurred between 4 and 5 weeks post-operation. The relatively swift pace in return to work and recreational activities is consistent with the minimally invasive nature of the hemiarthroplasty procedure and preservation of joint motion. Both these factors contribute to faster functional recovery compared to arthrodesis [11]. Patients in sedentary occupations came back to work within two to three weeks, whereas those in more physically demanding professions took up to six weeks before returning to full operation. The cohort included no cases of deep vein thrombosis or major wound complications requiring return to theatre.

Radiological assessment at final follow-up showed stable implant positioning generally, and in most cases, with no evidence of progressive subsidence or periprosthetic lucency. Mild radiographic changes consistent with adjacent joint degeneration were observed in some patients but did not correlate with clinical presentation or functional impairment.

## 4. Discussion

Arthritis of the first metatarsophalangeal joint can lead to stiffness and pain, significantly limiting mobility and affecting overall quality of life. When conservative treatments prove ineffective, surgical intervention may be necessary. The two standard surgical options for end-stage hallux rigidus are arthroplasty and arthrodesis. Arthroplasty aims to preserve joint motion, while arthrodesis fuses the joint to eliminate pain, resulting in a permanent loss of mobility.

Both procedures are widely performed, yet there is no consensus in the literature regarding the optimal treatment for hallux rigidus. Some surgeons opt for arthrodesis for its predictable pain relief, while others prefer arthroplasty to maintain movement. Studies indicate that both options have similar complication rates, with arthrodesis still regarded as the gold standard treatment for arthritic first metatarsophalangeal (MTP) joints [12,13]. Opting for arthrodesis or arthroplasty should be based on patient-specific factors such as age, activity levels, occupational and recreational levels. It should also take patient expectations regarding mobility into account and any presence of co-morbidities that may influence bone quality or healing ability. Younger and more active patients who wish to maintain the mobility of the first MTP joint to participate in sports or whose occupation demands frequent squatting or kneeling may benefit more from hemiarthroplasty. Conversely, in elderly patients with lower functional demands, or those with severe deformation or severe comorbidities, satisfactory results and pain relief may be achieved from arthrodesis. Shared decision making is therefore essential including having a realistic discussion over the perceived and actual benefits versus risks and limitations of each procedure to ensure that the selected treatment is specific to the individual patient’s goals and lifestyle.

Several studies have reported statistically significant improvements in patient-reported outcome measures after arthroplasty, including pain reduction and joint mobility improvements. Arthroplasty has been shown to restore mobility and decrease pain, enhancing physical and social functioning.

Maintaining a natural gait is a key advantage of arthroplasty compared to arthrodesis. While arthrodesis eliminates pain, it restricts patients’ ability to perform daily tasks like walking or engaging in sports due to the loss of joint motion. In contrast, arthroplasty allows patients to retain their range of motion, leading to improved functional outcomes and higher satisfaction rates [14,15].

Biomechanical studies have demonstrated that loss of motion at the first MTP joint following arthrodesis alters gait mechanics, with patients adopting compensatory mechanisms such as increased external rotation of the foot, decreased push-off power and changes in loading patterns across the midfoot and lesser toes. These compensations can later lead to progressive degeneration of the adjacent joints, including the interphalangeal joint of the great toe and lesser metatarsophalangeal joints. By preserving mobility of the first MTP joint, hemiarthroplasty can avoid these biomechanical compromises and may reduce the risk of long-term secondary joint degeneration. Patients in this cohort reported a more natural gait pattern. The preservation of dorsiflexion was especially valued for activities such as walking on uneven ground, climbing stairs, and even being able to wear a wide variety of footwear [16,17,18].

Despite its benefits, some patients report high revision rates after arthroplasty. However, these complications are often linked to surgical technique or implant quality issues rather than the procedure itself. It is expected that revision rates will decrease over time due to advancements in surgical equipment and increased surgeon experience. Indeed, the survivorship of arthroplasty implants is excellent at both 5 and 8 years, with only a small number of patients requiring revision surgery due to aseptic loosening [19,20,21].

Importantly, revision from hemiarthroplasty to arthrodesis remains a feasible option in the event of persistent pain, implant failure, or even the progression of degenerative change—unlike in total joint replacements of larger joints, where revision surgery can be technically complex with greater morbidity risks. Conversion of failed MTP joint hemiarthroplasty to arthrodesis is generally well tolerated, with most patients achieving satisfactory pain relief following revision [22]. The reversibility is an important consideration in the counselling process as it reassures both surgeon and patient that arthroplasty does not preclude future arthrodesis revision if required. In our cohort, four patients required revision to arthrodesis and were able to achieve satisfactory pain relief and return to normal activities of daily living, supporting the view that hemiarthroplasty can be regarded as a first-line option for joint preservation. The evolution of implants has played a crucial role in enhancing the outcomes of MTP joint replacement. Early attempts to replace the hallux metatarsophalangeal joint utilised metals and acrylics, which failed to provide long-term functional outcomes. This led to the introduction of first-generation silicone implants, which were initially used as single-stem implants and later as double-stemmed hinged designs. These innovations aimed to offer better joint mobility and patient satisfaction [23,24].

Silicone implants, also known as “dynamic spacers,” were developed to preserve joint space and restore motion to the MTP joint. Their effectiveness relied on the ingrowth of collagenous scar tissue to provide necessary alignment and support.

However, complications with silicone implants began to surface in the 1980s, tempering initial optimism. This prompted the introduction of second-generation titanium grommets to minimise wear from silicone implants. Despite these advancements, complications persisted, leading to a search for improved joint replacement options. Reported complications with silicone implants included synovitis, fragmentation of the implant material, and lymphadenopathy secondary to particle migration [25,26]. These issues led to a significant decline in the use of silicone implants in first MTP joint procedures. Although, they still continue to be utilised in some cases, notably in low-demand patients with rheumatoid arthritis. This resulted in the development of metal-on-polyethylene implants, a design that had proven successful for hip and knee replacements.

Early third-generation metal-on-polyethylene implants for the great toe faced limitations in range of motion and loosening. Subsequently, non-constrained versions were developed to address these issues, offering greater mobility and setting the standard for future implant advancements. Modern prosthetic options for the hallux MTP joint include silastic double-stemmed implants. Newer designs include advanced materials including pyro carbon, ceramic and highly cross-linked polyethylene to reduce wear, improve biocompatibility, and prolong implant longevity. The HemiCAP system employed in this study is a modern metallic hemiarthroplasty design that only resurfaces the metatarsal head [27]. This preserves the proximal phalangeal articular surface and minimises bone resection. This conservative approach is especially appealing for younger patients as it preserves bone stock and facilitates future revision surgery if required. Newer total joint systems, such as the Cartiva synthetic cartilage implant, have also emerged as promising alternatives to both arthrodesis and hemiarthroplasty, demonstrating promising results, although long-term comparison studies are required.

In this study, the results support the increasing body of literature that advocates for first metatarsophalangeal (MTP) joint arthroplasty as an effective alternative to arthrodesis for patients with hallux rigidus. The significant increase in AOFAS scores, from a preoperative mean of 35 to a postoperative mean of 88, demonstrates the considerable functional and symptomatic benefits of joint replacement in this patient group.

The findings are consistent with other studies that show arthroplasty preserves the range of motion while providing substantial pain relief, which is a crucial advantage over arthrodesis. The mean active and passive range of motion outcomes of 35 and 45 degrees, respectively, indicate that patients regained significant mobility after surgery, positively impacting their quality of life and ability to engage in daily activities [14,15].

However, the complication rates in this study, notably the 14.70% of patients who experienced postoperative stiffness, highlight the potential challenges of this procedure. Similar to other reports, the results show that 5.88% of patients required revision surgery due to persistent pain, reflecting the difficulties in achieving optimal long-term outcomes, particularly in managing postoperative complications such as stiffness and implant failure [27,28]. Postoperative stiffness is a well-known complication of first MTP joint hemiarthroplasty, which may be associated with inadequate intraoperative soft tissue release, insufficient cheilectomy, postoperative scarring and adhesion formation. Poor rehab compliance may also be a cause. Early and structured physiotherapy commencing within the first 48 h of surgery is critical to minimising the risk of stiffness.

Nevertheless, the study’s 94.12% implant survival rate at a maximum follow-up of 7 years is promising. This suggests that advancements in implant design and surgical techniques are likely to enhance the durability of MTP joint replacements. These results support the continued use of arthroplasty in carefully selected patients, especially those who prioritise maintaining joint motion over fusion.

In the present study, statistically significant improvement in postoperative AOFAS scores and range of motion was observed following metatarsophalangeal joint hemiarthroplasty (two-sided *p* < 0.05), supporting the growing body of evidence favouring joint-preserving surgery in appropriately selected patients.

Several limitations of this study should be acknowledged. The retrospective design introduces inherent risks of selection bias and incomplete data capture, although the standardisation of surgical technique and follow-up protocol mitigates some of this concern. Since a comparative arthrodesis group is not available, its absence limits direct comparison of outcomes between the two procedures. The use of AOFAS score as the main outcome measure has been criticised in the literature for its limited psychometric validation. Future studies would benefit from the inclusion of more rigorously validated patient-reported outcome measures, such as the Manchester–Oxford Foot Questionnaire or Foot Function Index. The single-surgeon, single-centre design may limit the extrapolation of findings to broader surgical practice. However, it also has the advantage of minimising variability in surgical technique and perioperative care. Future research should focus on randomised comparison studies between arthrodesis and hemiarthroplasty for end-stage hallux rigidus with long-term follow-ups (>10 years). Comparative studies between different implant designs (metal hemiarthroplasty, synthetic cartilage, total joint replacements) would also aid in informing patients on implant selection in clinical practice. Analyses comparing the cost-effectiveness of arthroplasty and arthrodesis, including indirect costs such as absence from work and impact on quality of life, would assist in evidence-based decision making at both clinical levels and in the context of policy.

## 5. Conclusions

In conclusion, long-term data shows that arthroplasty of the first metatarsophalangeal (MTP) joint is an effective surgical option for hallux rigidus. This procedure is linked to high patient satisfaction, improved range of motion, and considerable implant survival rates. Continued advances in implant design, refinement of surgical technique, and structured rehabilitation protocols are likely to further improve the long-term outcomes of this procedure. Further comparative studies, randomised controlled trials and long-term registry data will be essential to gain a clearer understanding of its overall efficacy within the surgical management of hallux rigidus and ensure that treatment decisions are informed by robust, patient-centred evidence.

## Figures and Tables

**Table 1:** Patient demographics and preoperative details.

Parameters	Results
Total patients (toes)	60 patients (68 toes)
Gender distribution	30 males, 30 females
Average age at surgery	49.1 years
Stage of Hallux Rigidus	Stage 2: 14; Stage 3: 38; Stage 4: 8

**Table 2:** Surgical outcomes.

Outcome Measures	Preoperative	Postoperative	*p*-Value
Active ROM	0–10 degrees	35 degrees	*p* < 0.05
Passive ROM	0–10 degrees	45 degrees	*p* < 0.05
AOFAS Score	35	88	*p* < 0.05
Patients with Post-op Stiffness (no pain)	-	14.70% (10 toes)	-
Patients Requiring Arthrodesis	-	5.88% (4 toes)	-
Survival Rate of Joint Replacement	-	94.12%	-

## Data Availability

The data presented in this study are available on request from the corresponding author.

## References

[1] Ho B., Baumhauer J. (2017). Hallux rigidus. EFORT Open Rev..

[2] Coughlin M.J., Shurnas P.S. (2003). Hallux rigidus: Demographics, etiology, and radiographic assessment. Foot Ankle Int..

[3] Sidon E., Rogero R., Bell T., McDonald E., Shakked R.J., Fuchs D., Daniel J.N., Pedowitz D.I., Raikin S.M. (2019). Long-term Follow-up of Cheilectomy for Treatment of Hallux Rigidus. Foot Ankle Int..

[4] Geldwert J.J., Rock G.D., McGrath M.P., Mancuso J.E. (1992). Cheilectomy: Still a useful technique for grade I and grade II hallux limitus/rigidus. J. Foot Surg..

[5] Roukis T.S. (2010). The need for surgical revision after isolated cheilectomy for hallux rigidus: A systematic review. J. Foot Ankle Surg..

[6] Kitaoka H.B., Alexander I.J., Adelaar R.S., Nunley J.A., Myerson M.S., Sanders M. (1994). Clinical rating systems for the ankle-hindfoot, midfoot, hallux, and lesser toes. Foot Ankle Int..

[7] SooHoo N.F., Vyas R., Samimi D. (2006). Responsiveness of the foot function index, AOFAS clinical rating systems, and SF-36 after foot and ankle surgery. Foot Ankle Int..

[8] Coster M.C., Rosengren B.E., Bremander A., Brudin L., Karlsson M.K. (2014). Comparison of the Self-reported Foot and Ankle Score (SEFAS) and the American Orthopedic Foot and Ankle Society Score (AOFAS). Foot Ankle Int..

[9] Ibrahim T., Beiri A., Azzabi M., Best A.J., Taylor G.J., Menon D.K. (2007). Reliability and validity of the subjective component of the American Orthopaedic Foot and Ankle Society clinical rating scales. J. Foot Ankle Surg..

[10] Baumhauer J.F., Nawoczenski D.A., DiGiovanni B.F., Wilding G.E. (2006). Reliability and validity of the American Orthopaedic Foot and Ankle Society Clinical Rating Scale: A pilot study for the hallux and lesser toes. Foot Ankle Int..

[11] Carpenter B., Smith J., Motley T., Garrett A. (2010). Surgical Treatment of Hallux Rigidus Using a Metatarsal Head Resurfacing Implant: Mid-term Follow-up. J. Foot Ankle Surg..

[12] Sanchez Guzman J., Gallo Oropeza R., Reyes Donado M., Martin Oliva X., Diaz Sanchez T. (2024). Arthrodesis vs arthroplasty for moderate and severe Hallux rigidus: Systematic review. Foot Ankle Surg..

[13] Voskuijl T., Onstenk R. (2015). Operative treatment for osteoarthritis of the first metatarsophalangeal joint: Arthrodesis versus hemiarthroplasty. J. Foot Ankle Surg..

[14] Beekhuizen S.R., Voskuijl T., Onstenk R. (2018). Long-term results of hemiarthroplasty compared with arthrodesis for osteoarthritis of the first metatarsophalangeal joint. J. Foot Ankle Surg..

[15] Mermerkaya M.U., Alkan E., Ayvaz M. (2018). Evaluation of metatarsal head resurfacing hemiarthroplasty in the surgical treatment of hallux rigidus: A retrospective study and mid-to long-term follow-up. Foot Ankle Spec..

[16] Rajan R.A., Kerr M., Hafesji-Wade A., Osler C.J., Outram T. (2024). A prospective clinical and biomechanical analysis of feet following first metatarsophalangeal joint arthrodesis for end stage hallux rigidus. Gait Posture.

[17] Stevens J., de Bot R.T.A.L., Hermus J.P.S., van Rhijn L.W., Witlox A.M. (2017). Clinical Outcome Following Total Joint Replacement and Arthrodesis for Hallux Rigidus: A Systematic Review. JBJS Rev..

[18] Gibson J.N., Thomson C.E. (2005). Arthrodesis or total replacement arthroplasty for hallux rigidus: A randomized controlled trial. Foot Ankle Int..

[19] Hilario H., Garrett A., Motley T., Suzuki S., Carpenter B. (2017). Ten-year follow-up of metatarsal head resurfacing implants for treatment of hallux rigidus. J. Foot Ankle Surg..

[20] Akcaalan S., Kavaklilar A., Caglar C., Simsek M.E., Gursoy S., Akkaya M. (2024). Long-term outcomes of first metatarsophalangeal hemiarthroplasty for hallux rigidus. Foot Ankle Surg..

[21] Circi E., Tuzuner T., Sukur E., Baris A., Kanay E. (2016). Metatarsal head resurfacing arthroplasty in the treatment of hallux rigidus: Is it reliable treatment option?. Musculoskelet. Surg..

[22] Garras D.N., Durinka J.B., Bercik M., Miller A.G., Raikin S.M. (2013). Conversion arthrodesis for failed first metatarsophalangeal joint hemiarthroplasty. Foot Ankle Int..

[23] Cracchiolo A., Weltmer J.B., Lian G., Dalseth T., Dorey F. (1992). Arthroplasty of the first metatarsophalangeal joint with a double-stem silicone implant. Results in patients who have degenerative joint disease failure of previous operations, or rheumatoid arthritis. J. Bone Jt. Surg. Am..

[24] Giza E., Sullivan M., Ocel D., Lundeen G., Mitchell M., Frizzell L. (2010). First metatarsophalangeal hemiarthroplasty for hallux rigidus. Int. Orthop..

[25] Bommireddy R., Singh S.K., Sharma P., El Kadafi M., Rajan D., Rowntree M. (2003). Long-term follow-up of silastic joint replacement of the first metatarsophalangeal joint. Foot.

[26] Majeed H. (2019). Silastic replacement of the first metatarsophalangeal joint: Historical evolution, modern concepts and a systematic review of the literature. EFORT Open Rev..

[27] Clement N.D., MacDonald D., Dall G.F., Ahmed I., Duckworth A.D., Shalaby H.S., McKinley J. (2016). Metallic hemiarthroplasty for the treatment of end-stage hallux rigidus: Mid-term implant survival, functional outcome and cost analysis. Bone Jt. J..

[28] Swanson A.B., Lumsden R.M., Swanson G.D. (1979). Silicone implant arthroplasty of the great toe. A review of single stem and flexible hinge implants. Clin. Orthop. Relat. Res..

